# Cytological basis and key metabolite profiling of fresh tobacco leaves with different oiliness indices in Yunyan 87

**DOI:** 10.3389/fpls.2026.1911537

**Published:** 2026-09-04

**Authors:** Xianfeng Hu, Wei Xu, Hao Chen, Shouhui Pan, Yuan Xue, Liyuan Zhang, Yubo Zhang

**Affiliations:** 1 College of Agriculture, Anshun University, Anshun, Guizhou, China; 2 Raw Material Supply Center, China Tobacco Guizhou Industrial Co., Ltd., Guiyang, Guizhou, China; 3 Technology Center, Guizhou Provincial Tobacco Company Anshun Branch, Anshun, Guizhou, China

**Keywords:** cultivation regimes, hexadecanamide, metabolomics, oleic acid, relative oiliness index, ultrastructure

## Abstract

**Introduction:**

Tobacco oiliness index is a key chemical trait that significantly influences tobacco quality.

**Methods:**

To elucidate the cytological features and key metabolites underlying varying relative oiliness indices, fresh Yunyan 87 tobacco leaves were analyzed by microscopic and metabolomic profiling.

**Results:**

The results indicated that leaves with high oiliness exhibited a tighter tissue architecture, characterized by closely packed mesophyll cells and reduced intercellular spaces; conversely, leaves with low oiliness showed a looser arrangement. The levels of cembratriene-diols, methyl nonanoate, and methyl palmitate in trichome exudates were markedly elevated in high-oil leaves compared with those in low-oil leaves. High-oil leaves exhibited a significantly greater abundance of starch grains and osmiophilic granules. In contrast, low-oil leaves displayed localized chloroplast swelling and mild mitochondrial vacuolation, suggesting insufficient or imbalanced accumulation of intracellular storage substances. The concurrent elevation of oleic acid, a signature lipid metabolite, exhibited a strong positive correlation with relative oiliness index and holds promise as a reliable biomarker for identifying and selecting high-oil tobacco leaves.

**Discussion:**

These findings offer a theoretical reference for the targeted breeding and agronomic management of high-oil tobacco varieties.

## 1 Introduction

Tobacco (*Nicotiana tabacum* L.) constitutes a pivotal economic crop, contributing substantially to the agricultural economies of numerous countries worldwide (Li et al., 2021; Xiao et al., 2025; Lei et al., 2025). The intrinsic quality traits of tobacco leaves serve as key determinants of both sensory evaluation outcomes and the industrial utilization of cigarette products (Liu et al., 2022; Ahmed and Hameed, 2024). Among these traits, leaf oiliness acts as a crucial chemical determinant closely associated with tobacco aroma, flavor profiles, and processing performance. At the macroscopic level, leaves with adequate oiliness display a plump, turgid appearance and an oily sheen under optimal moisture conditions, often feeling slightly greasy upon tactile examination. In contrast, oil-deficient leaves typically appear dry, lusterless, and even brittle (Hu et al., 2025). Oil-rich tobacco leaves are widely regarded as superior in quality, characterized by a more intense aroma, well-balanced taste, and enhanced processing suitability (Kelimujiang et al., 2024; Chen et al., 2025a). Volatile oils function as important aroma precursors; accordingly, high-oil leaves tend to emit a strong, refined aroma with minimal off-odors. Non-volatile oils enhance the smoking quality of tobacco by modulating its combustion behavior (Wang et al., 2025a). Furthermore, adequately oil-enriched leaves possess high elasticity and excellent filling capacity, burning uniformly and yielding cohesive ash that resists fragmentation, thereby improving their overall suitability for cigarette manufacturing. Nevertheless, the cytological basis and key metabolic pathways underlying oil accumulation in fresh tobacco leaves with contrasting relative oiliness index remain largely unexplored.

The metabolome comprises the entire complement of small-molecule metabolites within an organism, encompassing the chemical entities involved in cellular regulatory processes and their dynamic alterations (Maia et al., 2023; Mok et al., 2024). As a cornerstone of systems biology, metabolomics harnesses high-throughput analytical platforms to enable comprehensive qualitative and quantitative profiling of these metabolites, thereby unraveling metabolic networks and their intricate relationships with genetic and environmental factors (Ivanisevic and Sewduth, 2023; Chen et al., 2025b). In contrast to genomics and transcriptomics, the metabolome offers a global snapshot of the biological state, faithfully mirroring the downstream consequences of gene expression and protein activity, and thereby addressing fundamental gaps in our understanding of phenotypic variation (Silva et al., 2021; Shen et al., 2023). Recent multi-omics studies have highlighted the pivotal role of microbial communities in determining tobacco quality. Integrated non-targeted metabolomics and metagenomic profiling have demonstrated that microbial metabolic activities orchestrate the accumulation of aroma precursors in both mild- and full-flavored tobacco varieties (Jia et al., 2025). Similarly, during the stacking fermentation of cigar tobacco leaves (CTLs), complementary multi-omics strategies have revealed that microbial consortia govern the biosynthesis of volatile flavor compounds (VFCs) (Wu et al., 2023). Collectively, multi-omics technologies encompassing metabolomics, microbial ecology, and proteomics have elucidated the complex regulatory networks underlying tobacco quality formation, thereby laying a solid theoretical foundation for targeted trait improvement and precision processing (Wang et al., 2025b).

This study combined electron microscopy and untargeted metabolomics to systematically characterize the microstructural features and metabolite profiles of fresh tobacco leaves with varying oiliness indices. It aims to reveal the cytological and metabolic basis associated with oil accumulation in tobacco leaves under distinct cultivation regimes, providing a reference for the targeted breeding and cultivation optimization of high-oil tobacco.

## 2 Materials and methods

### 2.1 Experimental materials

Electron microscopy fixatives and PBS were obtained from Servicebio (Wuhan, China). Absolute ethanol and isoamyl acetate were supplied by Sinopharm Chemical Reagent Co., Ltd. (Shanghai, China), and osmium tetroxide (OsO_4_) by Ted Pella Inc. (Redding, CA, USA).

### 2.2 Tobacco cultivation

Field experiments were conducted in Niebulong Village, Maoying Town, Ziyun County, Anshun City, Guizhou Province, China (25.952456° N, 106.131907° E). The widely cultivated flue-cured tobacco variety Yunyan 87 was used as the test material. In this study, high-oil and low-oil treatments were established to simulate two typical cultivation management regimes in the tobacco-growing regions of Guizhou. For the high-oil treatment, a total of 6.0 kg pure nitrogen per thousand plants was applied, supplied by Guizhou Ketai Jinfu Agricultural Development Co., Ltd. This was combined with commercial organic fertilizer (Guizhou Guifu Ecological Fertilizer Co., Ltd.) containing 20% oil cake at 80 kg per thousand plants, decomposed oil cake (Guizhou Bailing Pharmaceutical & Fertilizer Factory) at 30 kg per thousand plants, and calcium magnesium phosphate fertilizer (Guizhou Ketai Jinfu Agricultural Development Co., Ltd.) at 25 kg per thousand plants. For the low-oil treatment, the nitrogen application rate was increased to 6.5 kg per thousand plants, supplied by the same manufacturer, and supplemented with oil cake–free commercial organic fertilizer (Guizhou Guifu Ecological Fertilizer Co., Ltd.) at >80 kg per thousand plants and calcium magnesium phosphate fertilizer (Guizhou Ketai Jinfu Agricultural Development Co., Ltd.) at 25 kg per thousand plants.

### 2.3 Relative oiliness index assessment of fresh tobacco leaves

The relative oiliness index of fresh tobacco leaves was determined using a handheld oil detection pen (Shenzhen Fuhuitong Technology Co., Ltd., China). The percentage data from the handheld oil detection pen represent the relative oiliness index, rather than the total lipid content determined by chemical extraction methods. The precision of the device was validated by performing ten consecutive measurements of the same sample under identical experimental conditions. For each tobacco grade, six parallel measurements were conducted. The precision was expressed as the relative standard deviation (RSD%), and the validation data are provided in **Supplementary Table 1**. Assessments were performed on the mid-lamina region of leaves exhibiting typical maturity characteristics, including partial chlorophyll degradation (chlorosis), approximately 70% yellowing, whitening of the main vein tips, and slight leaf curvature. Based on the measured values, samples were classified into a high-oil group (H; relative oiliness index ≥51%, characterized by pronounced oiliness) and a low-oil group (L; relative oiliness index <46%, showing minimal visible oiliness). Six biological replicates were included for each group.

### 2.4 Microstructural and ultrastructural observation of fresh tobacco leaves

#### 2.4.1 Scanning electron microscopy observation

Sampling and primary fixation: Fresh leaves of Yunyan 87 with high (H) and low (L) oiliness were excised into small tissue blocks (≤3 mm²). Sample preparation was carried out following the protocol described by Shar et al. (2024). Tissues were immediately fixed in electron microscopy fixative for 2 h at room temperature and subsequently stored at 4 °C. After gentle rinsing with PBS, samples were transferred to fresh fixative and subjected to secondary fixation at room temperature for 2 h, followed by storage at 4 °C. Post-fixation: Fixed samples were washed three times with 0.1 M phosphate buffer (PB, pH 7.4) for 15 min each. Dehydration: Samples were dehydrated through a graded ethanol series (30%, 50%, 70%, 80%, 90%, and 95%) for 15 min per step, followed by two changes of absolute ethanol (100%) for 15 min each, and then immersed in isoamyl acetate for 15 min. Drying and conductive coating: Samples were dried using a critical point dryer, mounted onto conductive carbon tape, and sputter-coated with gold for approximately 30 s using an ion sputter coater. Finally, microstructural images were acquired using a scanning electron microscope (SEM). The densities of glandular trichomes and stomata were quantified from SEM images using ImageJ software (Version 1.53e, NIH, USA). Pixel calibration was performed based on the scale bar. A standardized region with a fixed area (A) was selected at the center of each field of view. Only glandular trichomes with intact basal structures and stomata with clearly defined guard cell contours were counted (N). Density was calculated using the formula Density=N/A. For each sample, at least five random fields of view were analyzed, and the mean density was reported.

#### 2.4.2 Transmission electron microscopy observation

Sampling and primary fixation: Fresh leaves of Yunyan 87 with high (H) and low (L) oiliness were excised into small tissue blocks. Sample preparation was carried out following the method described by Chang et al. (2024). Tissue blocks were transferred into eppendorf tubes containing fresh electron microscopy fixative and subjected to vacuum infiltration to remove air bubbles until the tissues sank. Samples were fixed at room temperature for 2 h and subsequently stored and transported at 4 °C. Washing and post-fixation: Samples were washed three times with 0.1 M phosphate buffer (PB, pH 7.4) for 15 min each. For post-fixation, tissues were immersed in 1% osmium tetroxide (OsO_4_) prepared in 0.1 M PB (pH 7.4) and incubated in the dark at room temperature for 7 h. After fixation, samples were washed again three times with 0.1 M PB (pH 7.4) for 15 min each. Dehydration and infiltration: Samples were dehydrated through a graded ethanol series (30%, 50%, 70%, 80%, and 95%) for 1 h per step, followed by two exchanges of absolute ethanol (100%). Subsequently, samples were infiltrated with Spurr’s resin using a graded acetone-resin series: acetone:Spurr’s resin (3:1) at 37 °C for 2–4 h, (1:1) at 37 °C overnight, (1:3) at 37 °C for 2–4 h, and pure resin at 37 °C for 5–8 h. Embedding and polymerization: Samples were embedded in fresh Spurr’s resin within embedding molds and incubated at 37 °C overnight. Polymerization was performed at 60 °C for 48 h. The hardened resin blocks were then removed from the molds and stored prior to sectioning. Sectioning and staining: Ultrathin sections (60–80 nm) were cut using an ultramicrotome and collected on copper grids. Sections were stained with 2% uranyl acetate in absolute ethanol (protected from light) for 8 min. Grids were examined under a transmission electron microscope, and digital micrographs were acquired for subsequent analysis. The densities of osmiophilic granules and lipid droplets were quantified from TEM images using ImageJ software (Version 1.53e, NIH, USA). Pixel calibration was performed based on the scale bar (Set Scalefunction). A standardized region with a fixed area (A) was selected at the center of each field of view. Both osmiophilic granules and lipid droplets within the defined area were enumerated (N). Density was calculated according to the formula Density=N/A. For each sample, data were derived from at least five independent fields of view and expressed as the mean value.

### 2.5 Determination of secretion contents in fresh tobacco leaves

#### 2.5.1 Determination of cembratrienediol content

A series of standard working solutions of cembratrienediol were prepared by diluting the stock solution in 70% acetonitrile to final concentrations of 1.00, 0.50, 0.25, 0.125, and 0.0625 mg·mL^-^¹. Fresh tobacco samples were accurately weighed and transferred into 15 mL centrifuge tubes fitted with polytetrafluoroethylene (PTFE) caps. Each sample was extracted twice with 10 mL of n-hexane under ultrasonication at 30 °C for 20 min (45 kHz). After cooling to room temperature, the extracts were centrifuged at 3000 × gfor 10 min. The pooled supernatants were evaporated to dryness under a gentle nitrogen stream and reconstituted in 1 mL of mobile phase. Prior to instrumental analysis, the solutions were filtered through a 0.22 μm membrane filter. Chromatographic separation was performed on a Thermo C_18_ column (2.1 mm × 50 mm) using 70% acetonitrile as the mobile phase under isocratic elution at a flow rate of 0.3 mL·min^-^¹. The column temperature was maintained at 35 °C, and detection was carried out at 200 nm.

#### 2.5.2 Determination of methyl nonanoate and methyl palmitate

Standard stock solution: A stock solution of the reference standard was prepared by dissolving an appropriate amount in n-hexane and diluting to a final volume of 10 mL with n-hexane. The solution was stored in a sealed container at 0-4 °C. Working standard solutions were freshly prepared by serial dilution of the stock solution in n-hexane, yielding concentrations of 2.5, 5, 10, 50, and 200 μg·mL^-^¹. Sample preparation: (1) Weighing: Approximately 0.1-1.0 g of sample was accurately weighed into a 250 mL flat-bottom flask. (2) Acid hydrolysis: Five milliliters of hydrochloric acid solution was added, and the mixture was homogenized thoroughly. The flask was incubated in a water bath at 70-80 °C for 40 min. (3) Lipid extraction: After hydrolysis, 5 mL of 95% ethanol was added and mixed well. The extraction was repeated three times. The combined extracts were transferred to a separatory funnel, rinsed with a diethyl ether–petroleum ether mixture (1:1, v/v), and the organic layers were collected in a 250 mL flask. Solvents were removed by rotary evaporation to obtain the crude lipid residue. (4) Saponification and methylation: To the lipid residue, 4 mL of 2% NaOH in methanol was added, and the mixture was refluxed at 80 ± 1 °C using a condenser until oil droplets disappeared completely. Subsequently, 3.5 mL of 15% BF_3_ in methanol was introduced through the condenser, and refluxing was continued for 2 min. After cooling, 7.5 mL of n-heptane was added, and the mixture was shaken vigorously for 2 min. Saturated NaCl solution was then added to promote phase separation, and the upper organic layer was collected for analysis. GC analytical conditions: Analysis was performed on a gas chromatograph equipped with a CD-2560 capillary column, using nitrogen as the carrier gas. The injector temperature was set at 250 °C with a split ratio of 1:10. The oven temperature was programmed as follows: initial hold at 140 °C for 5 min, ramped to 240 °C, and held for 30 min.

### 2.6 Metabolomics analysis

#### 2.6.1 Sample preparation

Sampling was restricted to the middle leaves (the 9th leaf from the stem base). Leaves were collected at physiological maturity, identified by distinct decolorization, approximately 70% yellowing, bleached main vein apices, and incipient marginal curling. A single functional leaf was harvested per plant. Biological replicates (n=6) consisted of composite samples prepared by mixing five randomly selected plants from the same experimental plot. Samples were immediately snap-frozen in liquid nitrogen and stored at −80 °C until further analysis. High- and low-oil samples were designated as M_H_1 to M_H_6 and M_L_1 to M_L_6, respectively. All samples were transported on dry ice to Shenzhen Weikemeng Technology Group Co., Ltd. for untargeted metabolomic profiling.

#### 2.6.2 Metabolites extraction

Tissues (100 mg) were individually ground in liquid nitrogen. The homogenates were resuspended in pre-chilled 80% methanol and vortexed thoroughly. After incubation on ice for 5 min, samples were centrifuged at 15,000 × g at 4 °C for 20 min. An aliquot of the supernatant was diluted with LC-MS grade water to a final methanol concentration of 53%. The samples were then transferred to fresh Eppendorf tubes and centrifuged again under the same conditions (15,000 × g, 4 °C, 20 min). Finally, the supernatants were collected and injected into an LC-MS/MS system for analysis.

#### 2.6.3 UHPLC-MS/MS analysis

UHPLC-MS/MS analyses were performed using a Vanquish UHPLC system (Thermo Fisher Scientific, Germany) coupled to an Orbitrap Q Exactive™ HF or Q Exactive™ HF-X mass spectrometer (Thermo Fisher Scientific, Germany). Chromatographic separation was conducted on a Hypersil GOLD column (100 × 2.1 mm i.d., 1.9 μm) at a flow rate of 0.2 mL/min with a 12-min gradient elution. The mobile phases consisted of 0.1% formic acid in water (eluent A) and methanol (eluent B). The gradient program was set as follows: 0–1.5 min, 2% B; 1.5–4.5 min, 2%–85% B; 4.5–10 min, 85%–100% B; 10–10.1 min, 100%–2% B; followed by re-equilibration at 2% B until 12 min. Mass detection was carried out in both positive and negative ionization modes. The optimized source parameters were as follows: spray voltage, ± 3.5 kV; capillary temperature, 320 °C; sheath gas flow rate, 35 arb; auxiliary gas flow rate, 10 L/min; S-lens RF level, 60; and auxiliary gas heater temperature, 350 °C.

#### 2.6.4 Data processing and metabolite identification

Raw data files (.raw) were imported into Compound Discoverer 3.3 (CD 3.3) for preprocessing. Initial filtration was performed based on retention time, mass-to-charge ratio (m/z), and other parameters for each metabolite. Peak area normalization against the first quality control (QC1) sample was then conducted to improve identification accuracy. Parameters including a mass deviation tolerance of 5 ppm, signal intensity deviation of 30%, minimum signal intensity threshold, and adduct ion types were configured for peak extraction and peak area-based quantification. Target ions were subsequently integrated, followed by molecular formula prediction via analysis of precursor and fragment ions. Putative identifications were matched against three spectral libraries: mzCloud (https://www.mzcloud.org/), mzVault, and Masslist. Background ions were removed using blank samples. Metabolites with coefficient of variation (CV) of relative peak areas exceeding 30% in QC samples were excluded. The final dataset comprised confirmed metabolite identifications and corresponding relative quantitative results. All data processing procedures were implemented on a Linux operating system (CentOS version 6.6) using R and Python; detailed information regarding package versions and software specifications is provided in the readmefile accompanying the result dataset.

### 2.7 Statistical analysis

Raw mass spectrometry data were processed sequentially through peak detection, alignment, and normalization. The resulting datasets underwent qualitative and quantitative annotation via matching against established metabolite databases and authentic standards. Subsequently, multivariate statistical analyses, specifically principal component analysis (PCA) and hierarchical clustering, were employed to identify metabolites exhibiting significant variation across tobacco samples of varying maturity. The data are expressed as mean ± standard deviation (SD). Differences among groups were assessed by one-way analysis of variance (ANOVA), followed by *Duncan*’s multiple range test for post hoc comparisons. Mean values followed by different lowercase letters indicate significant differences at *P* < 0.05. All statistical analyses were performed using SPSS 26.0 (IBM Corp., Armonk, NY, USA), and graphs were generated with Origin 2021 (OriginLab Corp., Northampton, MA, USA).

## 3 Results and discussion

### 3.1 Agronomic traits of fresh Yunyan 87 leaves in the field

**Figure 1:**
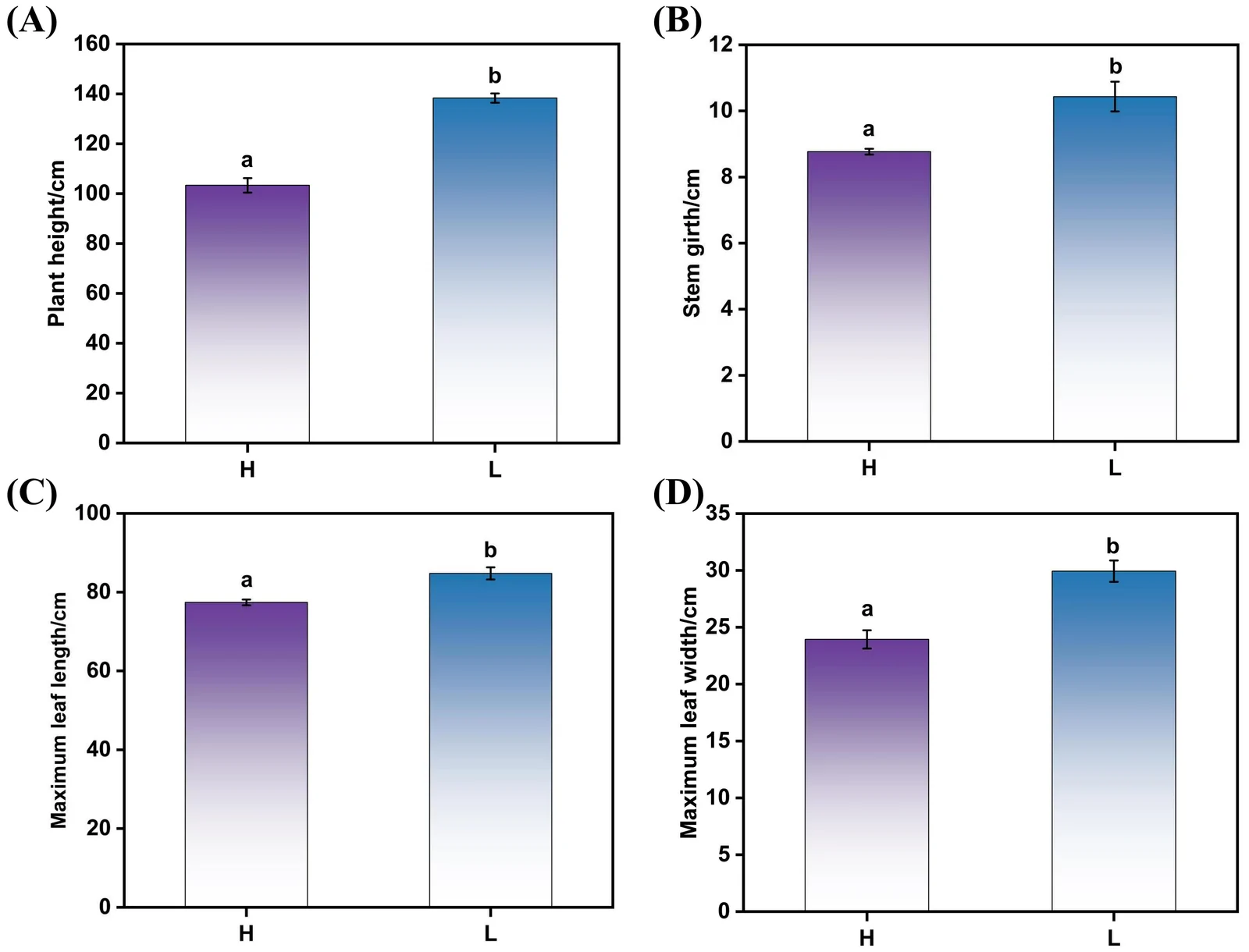
Agronomic traits of fresh Yunyan 87 tobacco leaves with different relative oiliness indices. **(A)** Plant height; **(B)** Stem girth; **(C)** Maximum leaf length; **(D)** Maximum leaf width. H, High-oil fresh leaves; L, Low-oil fresh leaves. Mean values followed by different lowercase letters are significantly different at *P* < 0.05 based on one-way ANOVA with *Duncan*’s multiple range test.

**Figure 2:**
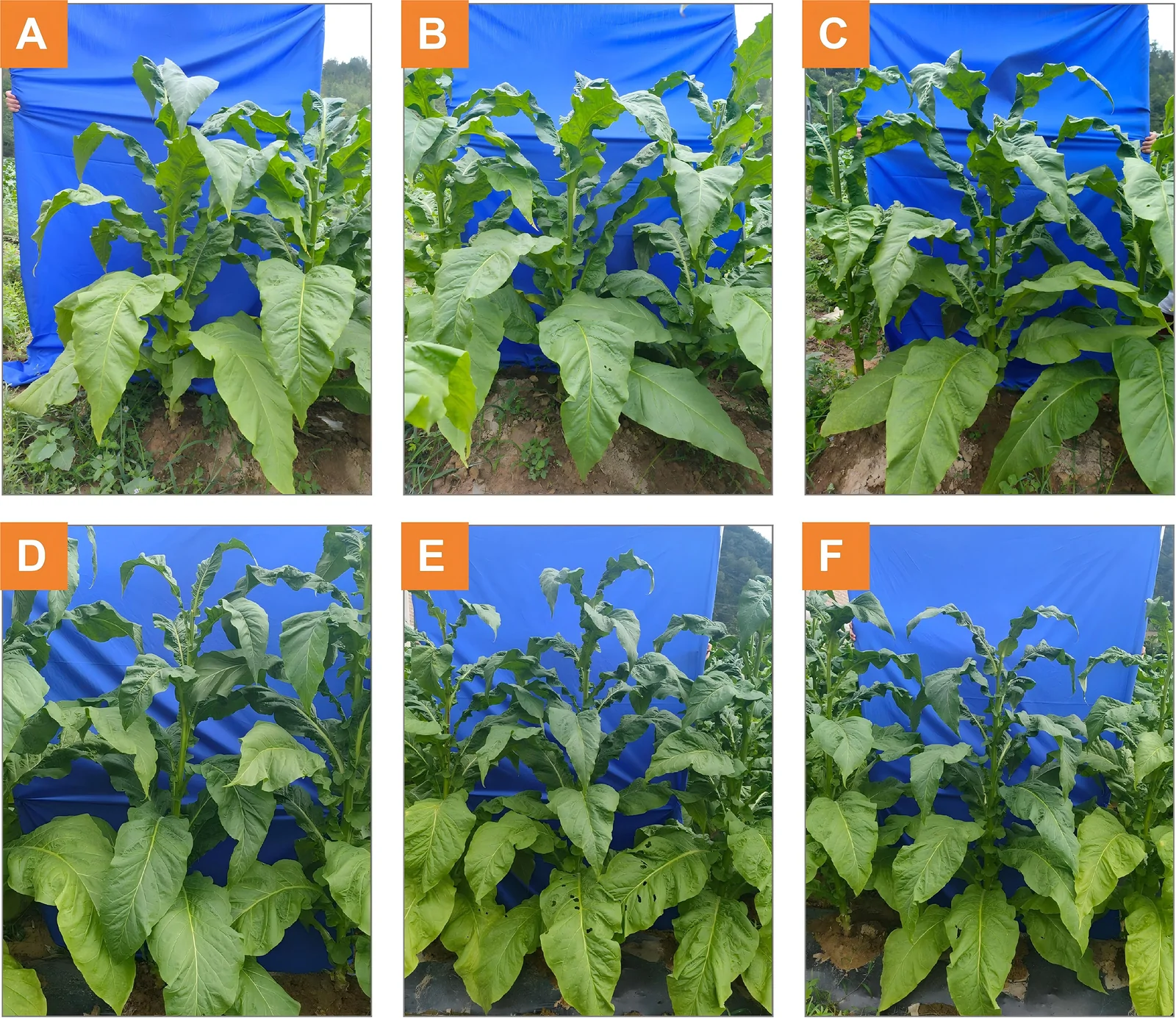
Field growth performance of fresh Yunyan 87 tobacco leaves with different relative oiliness indices. **(A–C)** High-oil fresh tobacco leaves; **(D–F)** Low-oil fresh tobacco leaves.

Agronomic traits, including plant height, stem girth, maximum leaf length, and maximum leaf width, were measured in Yunyan 87 plants differing in oiliness. Based on relative oiliness index, samples were classified into a high-oil group (Group H) and a low-oil group (Group L). All measured traits were significantly greater in Group L than in Group H (**Figure 1**). Specifically, mean plant height was 138.33 cm in Group L compared with 103.33 cm in Group H; mean stem girth was 10.43 cm and 8.76 cm, respectively; maximum leaf length averaged 84.76 cm and 77.40 cm; and maximum leaf width averaged 29.93 cm and 23.87 cm. Collectively, these findings indicate that high-oil tobacco plants display agronomic characteristics typical of medium-sized phenotypes under field conditions. Representative field performance is shown in **Figure 2**. Zhu et al. (2023) reported that nitrogen application significantly altered the expression levels of genes related to nitrogen uptake, assimilation, and translocation. This enhancement of carbon and nitrogen assimilation capacity boosted biomass and ultimately increased yield. Upon entering the reproductive growth stage, photosynthates are translocated to developing seeds for the synthesis of proteins and lipids. However, protein synthesis takes precedence over lipid synthesis and consumes substantial photosynthates, thereby constraining lipid biosynthesis. These results demonstrate clear phenotypic divergence across cultivation modes, with high-oil tobacco showing a dwarfed plant type. Nevertheless, because the high- and low-oil regimes involve concurrent variations in nitrogen rate, oil cake, and organic fertilizer composition, the observed phenotypic differences should be regarded as a holistic response to the overall cultivation mode, not the direct outcome of any single factor.

### 3.2 Microscopic morphology and cellular ultrastructure of fresh tobacco leaves

#### 3.2.1 Microscopic morphology of fresh Yunyan 87 tobacco leaves

**Figure 3:**
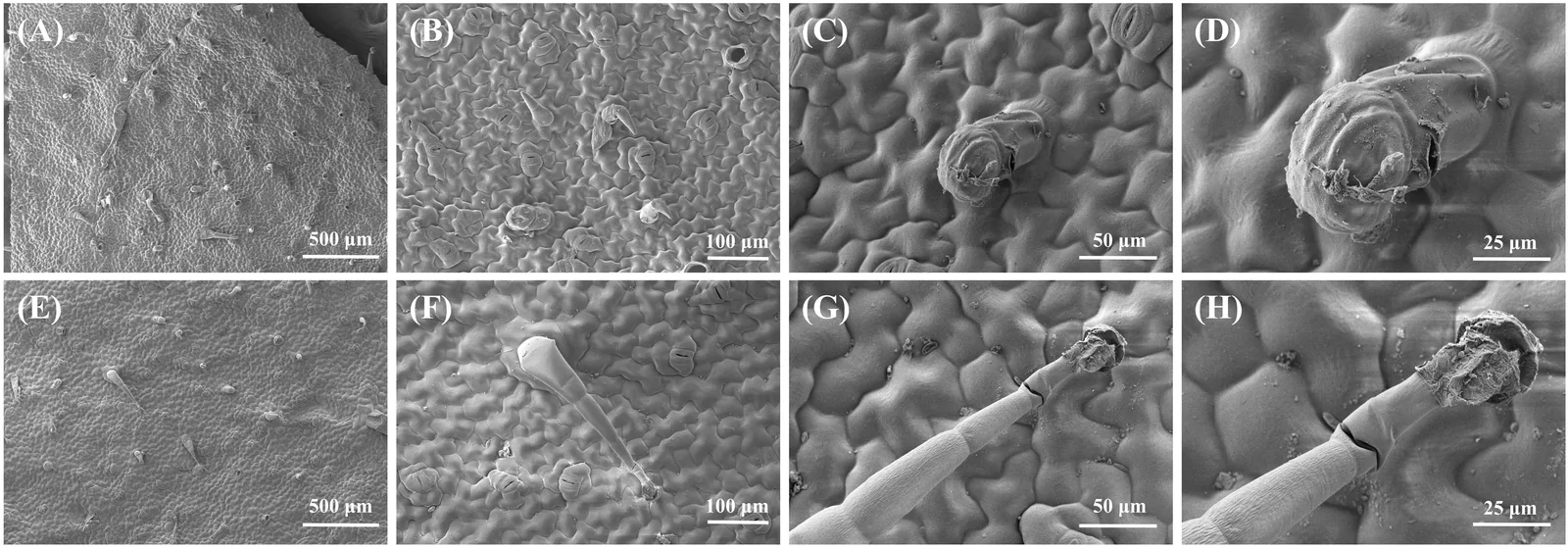
Microscopic morphology of fresh Yunyan 87 tobacco leaves with different relative oiliness indices. **(A–D)** High-oil fresh tobacco leaves; **(E–H)** Low-oil fresh tobacco leaves.

Tobacco glandular trichomes are specialized epidermal structures on leaf surfaces that play a pivotal role in determining leaf quality (Wang et al., 2022; Zhang et al., 2023). To characterize micromorphological variations associated with relative oiliness index, scanning electron microscopy (SEM) was employed to examine the epidermal microstructure of fresh tobacco leaves with high and low oiliness (**Figure 3**). Marked differences were observed in trichome morphology and secretion status. In high-oil leaves, glandular trichomes were densely distributed, with turgid head cells displaying well-defined outlines. Surfaces of these trichomes frequently exhibited secretory coatings or exudates, indicative of advanced developmental status and active secretion (**Figures 3A–D**). By contrast, low-oil leaves showed reduced trichome density. Most glandular heads were shriveled, collapsed, or entirely detached, leaving only denuded stalks. The lack of secretory deposits implied pronounced structural deterioration (**Figures 3E–H**). Quantitative analysis corroborated these morphological observations, revealing that the mean glandular trichome density in high-oil leaves was 10.98 trichomes/mm², significantly higher than the 6.49 trichomes/mm² recorded in low-oil leaves. This indicates a denser trichome distribution in high-oil tobacco. Furthermore, the density and secretory status of glandular trichomes are closely correlated with leaf oiliness; the dense and functionally active trichomes observed in high-oil leaves likely facilitate the synthesis and accumulation of oily exudates (**Supplementary Figure S1**). In addition, quantitative analysis revealed that the stomatal density in high-oil leaves averaged 47.12 stomata/mm², which was significantly higher than the 35.43 stomata/mm² observed in low-oil leaves, indicating more vigorous stomatal development and distribution in the former (**Supplementary Figure S2**). Such stomatal configurations likely enhance gas-exchange capacity, thereby promoting photosynthesis and the biosynthesis of compounds relevant to leaf quality. Glandular trichomes on the leaf surface serve as pivotal sites for the biosynthesis and secretion of key aroma compounds and lipid-derived constituents. The exudates of these trichomes are rich in terpenoids, fatty acids, and their derivatives, which constitute the primary source of leaf oiliness. Consequently, a higher density and enhanced secretory activity of glandular trichomes are typically associated with greater accumulation of leaf oils (Yang et al., 2025).

#### 3.2.2 Ultrastructure of Yunyan 87 fresh tobacco leaf cells

**Figure 4:**
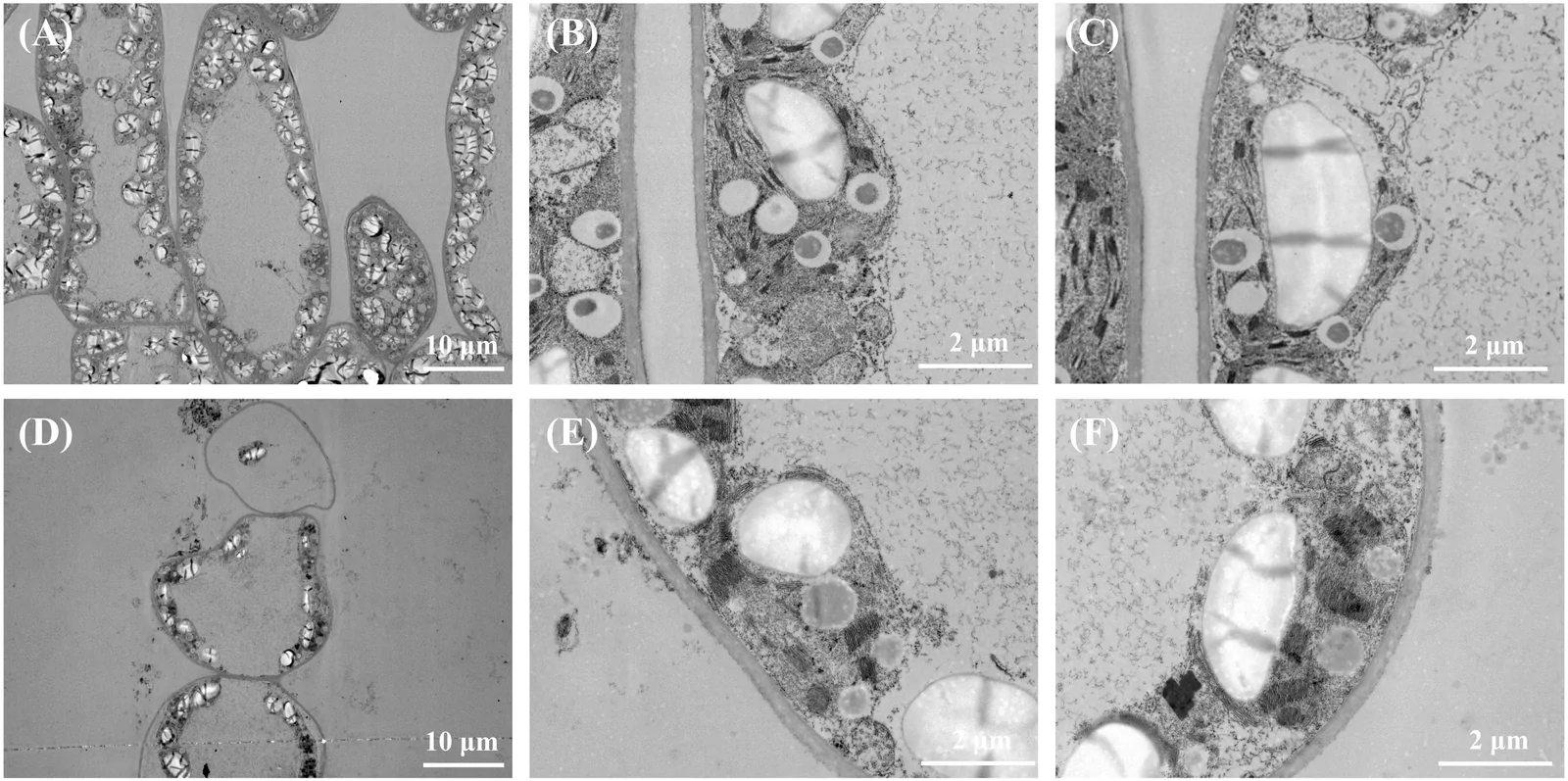
Ultrastructure of fresh Yunyan 87 tobacco leaves with different relative oiliness indices. **(A–C)** Fresh leaves with high oil; **(D–F)** Fresh leaves with low oil.

Transmission electron microscopy (TEM) was employed to investigate the cellular ultrastructure of fresh tobacco leaves with high and low oiliness (**Figure 4**). At the tissue level, mesophyll cells in high-oil leaves were tightly packed, with narrow intercellular spaces and clearly defined, intact cell walls, indicative of strong physiological activity and structural stability. By contrast, low-oil leaves exhibited a looser cellular arrangement, widened intercellular gaps, and localized cell wall shrinkage or deformation, suggesting comparatively limited water retention capacity and metabolic vigor. Pronounced differences were also evident in organelle development and storage product accumulation. High-oil samples (**Figures 4B, C**) contained abundant spherical, electron-dense lipid droplets and well-developed osmiophilic granules. These lipid reserves were relatively large, uniformly distributed, and coexisted with structurally intact organelles, including mitochondria and the endoplasmic reticulum, reflecting active biosynthetic and secretory functions. In low-oil samples (**Figures 4E, F**), however, the number and size of lipid droplets were markedly reduced, and their electron density was diminished. Ultrastructural quantitative analysis revealed that the densities of osmiophilic granules, lipid droplets, and starch grains were significantly higher in high-oil leaves than in low-oil leaves, with mean values of 0.12 *vs*. 0.05 granules/μm², 0.06 *vs*. 0.03 droplets/μm², and 0.05 *vs*. 0.02 grains/μm², respectively. These findings indicate a more substantial accumulation of storage substances and metabolites within the epidermal cells of high-oil tobacco, providing a cytological basis for its superior oiliness (**Supplementary Figure S3–5**). Partial organelle degradation and vacuolation were observed in certain areas, indicating limited lipid accumulation and compromised metabolic performance. Collectively, the compact cellular architecture, extensive lipid droplet deposition, and well-preserved organelle systems in high-oil leaves provide a structural foundation for their superior quality, shedding light on the subcellular mechanisms underlying the reduced oiliness of inferior leaves. As the primary carbohydrate storage form, starch grains undergo *Maillard* reactions to produce caramel and nutty aroma notes. Osmiophilic granules, defined as intracellular accumulations of lipophilic substances within chloroplasts, are rich in aroma precursors. These precursors are gradually released during curing alongside starch degradation, shaping the unique aromatic profile of the leaves (Wen et al., 2023).

### 3.3 Contents of related secretions in fresh Yunyan 87 tobacco leaves

#### 3.3.1 Content of cembratriendiols in fresh Yunyan 87 tobacco leaves

**Figure 5:**
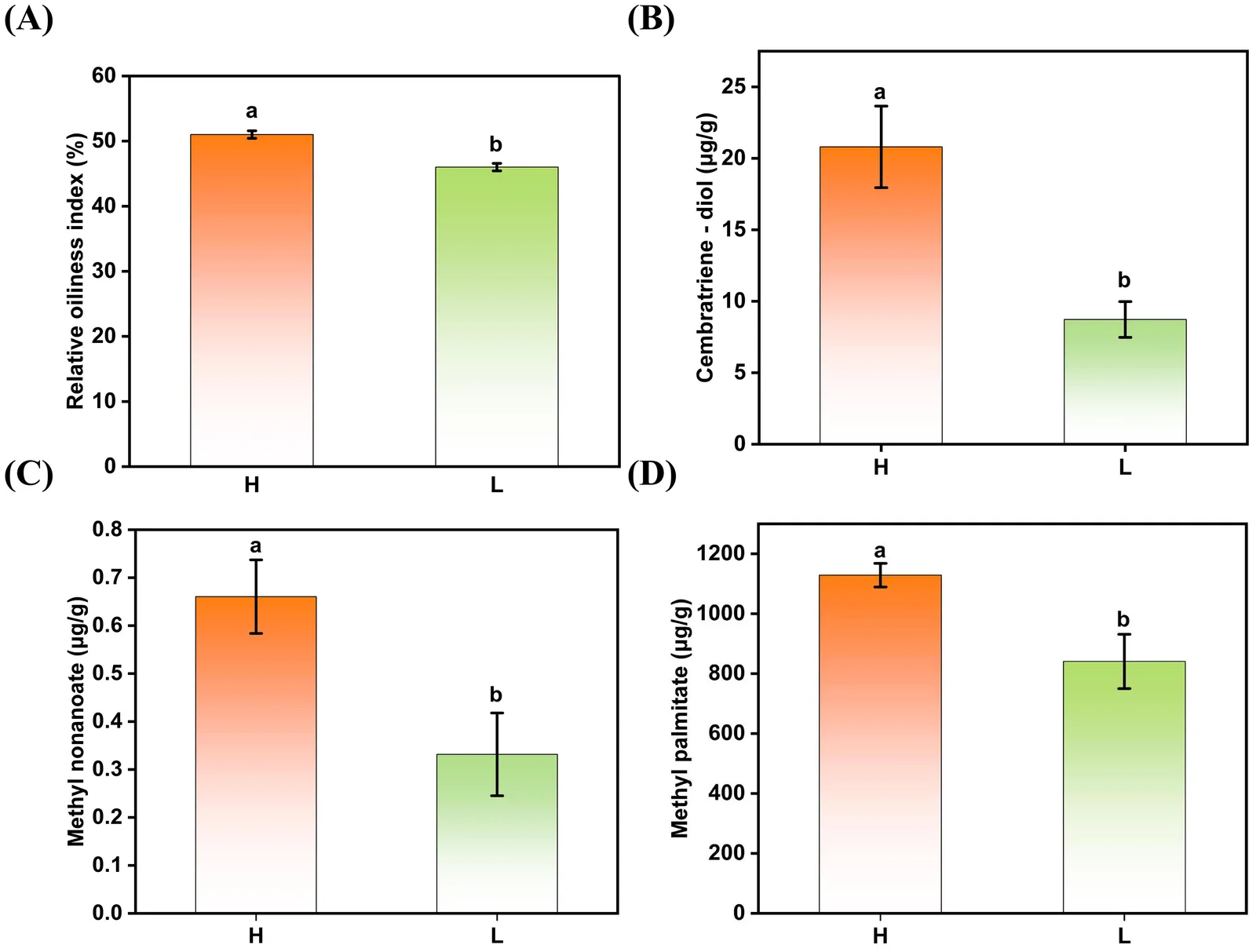
Contents of related metabolites in fresh Yunyan 87 tobacco leaves with different relative oiliness indices. **(A)** Relative oiliness index; **(B)** Cembratriendiol content; **(C)** Methyl nonanoate content; **(D)** Methyl palmitate content. H, High-oil fresh tobacco leaves; L, Low-oil fresh tobacco leaves. Mean values followed by different lowercase letters are significantly different at *P* < 0.05 based on one-way ANOVA with *Duncan*’s multiple range test.

Cembratriendiols are key cembranoid diterpenes in tobacco, predominantly accumulating in glandular trichome secretions and the cuticular wax layer (Liu et al., 2024). To investigate differences in cembratriendiol content between fresh leaves with contrasting oil levels, high-performance liquid chromatography (HPLC) was used to quantify these compounds in fresh leaves of the Yunyan 87 cultivar. As shown in **Figure 5B**, the cembratriendiol content in high-oil fresh leaves reached 20.81 μg/g, whereas that in low-oil fresh leaves was only 8.72 μg/g. These findings demonstrate that high-oil fresh tobacco leaves accumulate substantially higher levels of cembratriendiols than their low-oil counterparts.

#### 3.3.2 Content of methyl nonanoate in fresh Yunyan 87 tobacco leaves

Methyl nonanoate is a volatile aroma compound that contributes fruity, waxy, and faintly sweet notes to tobacco leaves (Sirilertpanich et al., 2024). To assess differences in methyl nonanoate content among fresh leaves with contrasting oil levels, high-performance gas chromatography (GC) was employed to quantify its concentration in fresh leaves of the Yunyan 87 cultivar. As shown in **Figure 5C**, the methyl nonanoate content in high-oil fresh leaves was 0.6604 μg/g, whereas that in low-oil fresh leaves was only 0.3315 μg/g. These findings demonstrate that high-oil fresh tobacco leaves accumulate markedly higher levels of methyl nonanoate than their low-oil counterparts. Furthermore, methyl palmitate, a lipophilic secondary metabolite in tobacco, plays a key role in leaf texture, combustibility, and aromatic expression (Fan et al., 2023).

#### 3.3.3 Content of methyl palmitate in fresh Yunyan 87 tobacco leaves

Methyl palmitate exhibits a faint waxy scent or is virtually odorless; however, it emits fatty and waxy aromas when combusted at elevated temperatures (Stöppelmann et al., 2023; Chu et al., 2023). To evaluate differences in methyl palmitate content among fresh leaves with contrasting oil levels, gas chromatography (GC) was used to quantify its concentration in fresh leaves of the Yunyan 87 cultivar. As shown in **Figure 5D**, the methyl palmitate content in high-oil fresh leaves reached 1128.54 μg/g, whereas that in low-oil fresh leaves was only 840.75 μg/g. These findings demonstrate that high-oil fresh tobacco leaves accumulate substantially higher levels of methyl palmitate than their low-oil counterparts.

### 3.4 Metabolomics data analysis

#### 3.4.1 Normalization and correction

**Figure 6:**
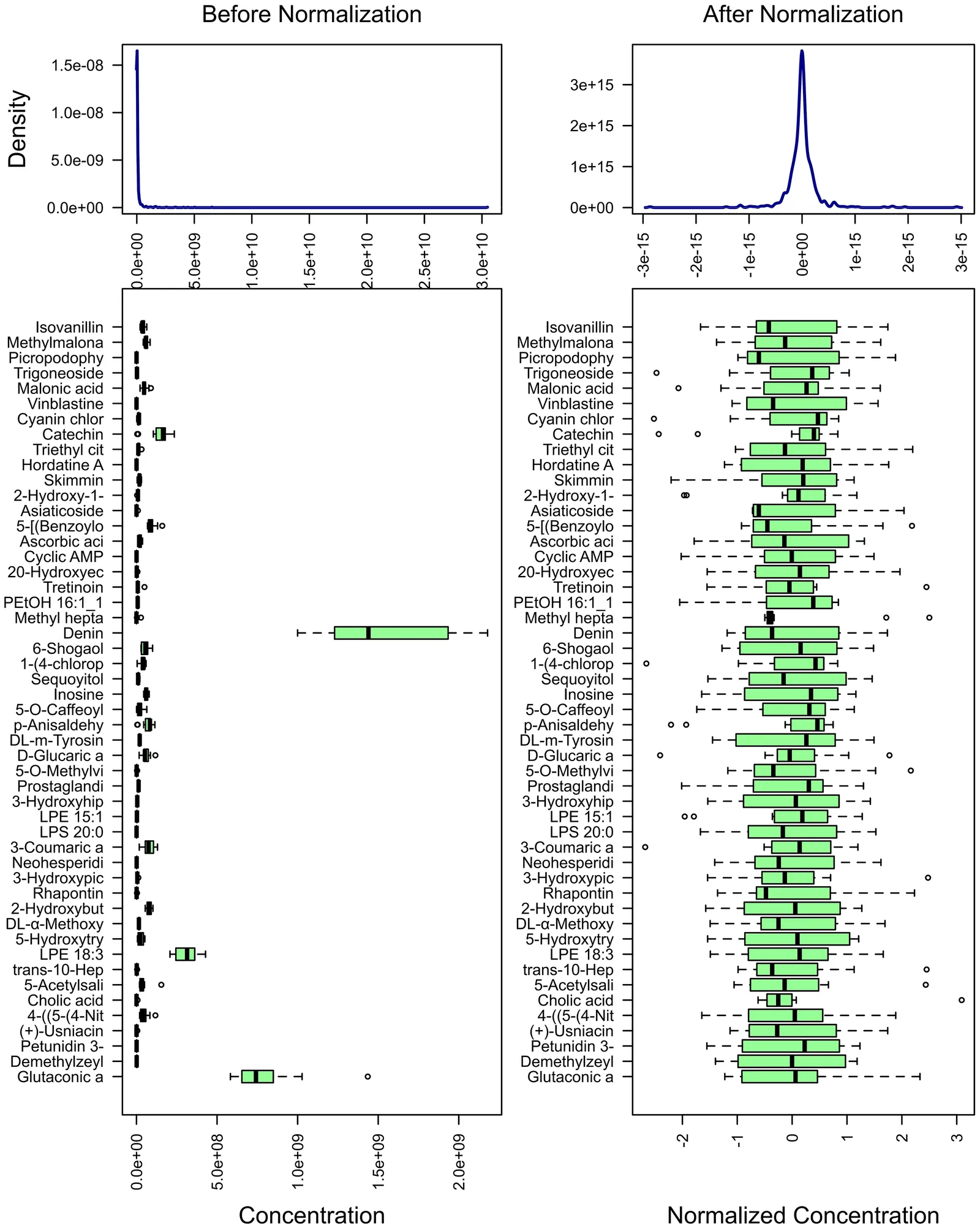
Comparison of metabolite distributions before and after normalization.

Normalization in metabolomics employs specialized statistical methods and algorithms to transform raw metabolomic data. Its primary purpose is to remove variations originating from non-biological sources, thereby improving dataset comparability and reliability while facilitating the accurate extraction of biological insights. As demonstrated in **Figure 6**, prior to normalization, the median and interquartile ranges of metabolite abundance levels displayed substantial variability. Following correction, however, these metrics converged to a consistent baseline.

#### 3.4.2 Quality assessment and quality control

**Figure 7:**
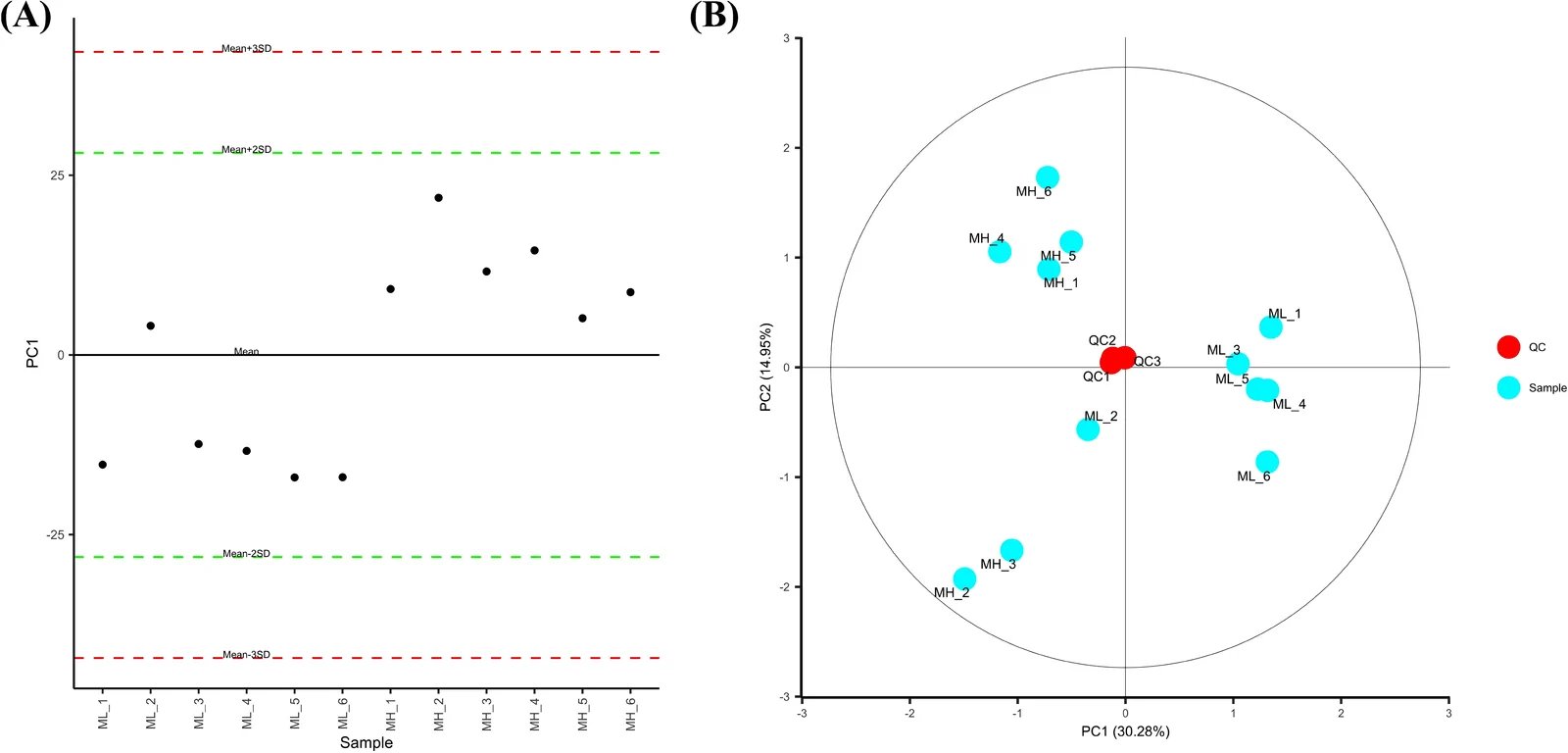
Distribution of samples along PC1 **(A)** and PCA score plot of quality control (QC) samples **(B)**. MH, High-oil fresh tobacco leaves; ML, Low-oil fresh tobacco leaves.

Quality control (QC) constitutes a critical component of bioanalytical assays, underpinning the reliability and accuracy of omics measurements. As shown in **Figure 7**, all data points remained within the control limits, with the majority fluctuating around the center line within two standard deviations, indicating satisfactory analytical reproducibility. In the PCA score plot, the QC samples formed a tight cluster, confirming stable instrument performance and excellent methodological robustness. In contrast, samples from different sampling sites exhibited a dispersed distribution and were distinctly separated from the QC cluster along the PC1 axis, highlighting significant chemical variability across the actual sample set.

#### 3.4.3 Unsupervised PCA analysis

**Figure 8:**
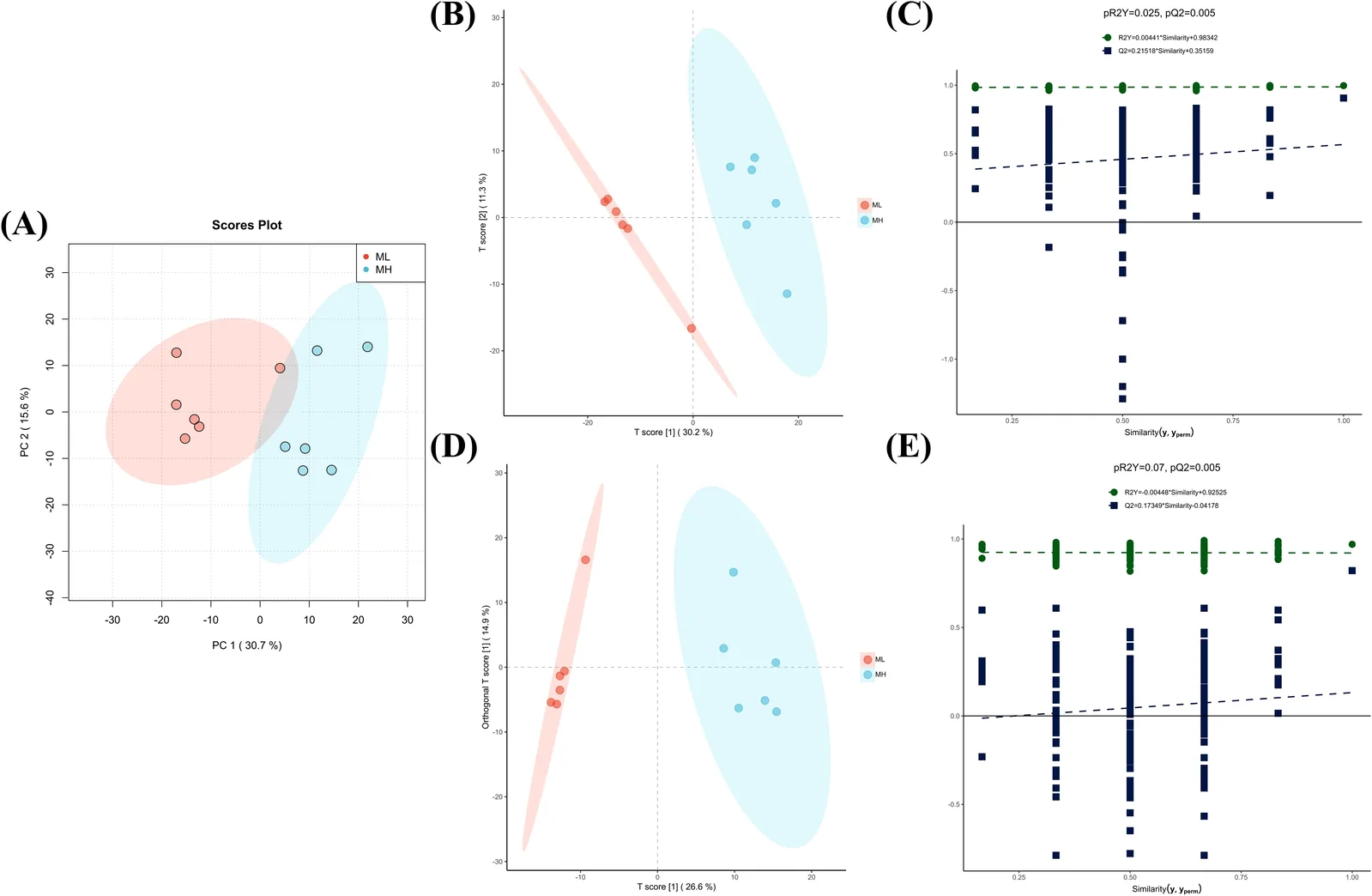
**(A)** PCA score plot; **(B)** PLS-DA scatter plot; **(C)** Permutation test plot for PLS-DA; **(D)** OPLS-DA scatter plot; **(E)** Permutation test plot for OPLS-DA. MH, High-oil fresh tobacco leaves; ML, Low-oil fresh tobacco leaves.

To evaluate overall metabolite variations among groups and variability within groups, principal component analysis (PCA) was conducted on the twelve samples. As shown in the score plots of PCA, PLS-DA, and OPLS-DA (**Figure 8**), the six biological replicates of high- and low-oil tobacco leaves formed distinct clusters, indicating minimal systematic error arising from experimental procedures or instrumental instability. In the PLS-DA model, R²X(cum) was 0.513, R²Y(cum) was 0.997, and Q²(cum) was 0.906. For the OPLS-DA model, R²X(cum) was 0.415, R²Y(cum) was 0.97, and Q²(cum) was 0.821. These results demonstrate good data reproducibility and confirm the reliability of the dataset for downstream analyses. Notably, samples with high and low oiliness were clearly separated in multidimensional space, revealing significant metabolic differences between tobacco leaves with distinct oil levels.

#### 3.4.4 Biological roles of differential metabolites

**Figure 9:**
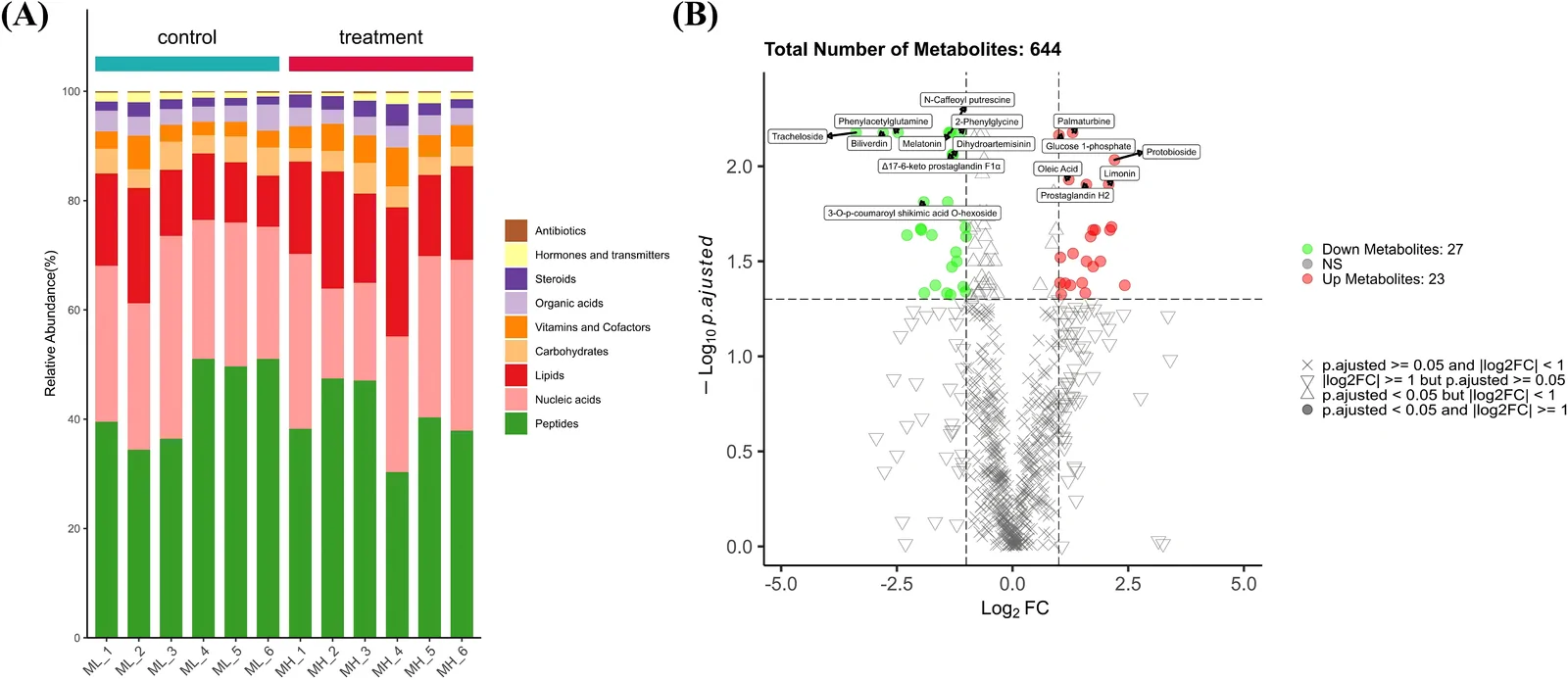
**(A)** Percentage of differential metabolites by biological role; **(B)** Volcano plot of differential metabolites. MH, High-oil fresh tobacco leaves; ML, Low-oil fresh tobacco leaves.

All identified metabolites were annotated using the KEGG database to assign biological functions. The relative contributions of these functional categories were subsequently calculated and visualized using a stacked column chart (**Figure 9A**). Major categories included antibiotics, hormones and transmitters, steroids, organic acids, vitamins and cofactors, carbohydrates, lipids, nucleic acids, and peptides. Notably, lipid levels were significantly elevated in high-oil fresh tobacco leaves compared to low-oil counterparts. Lipids function not only as essential constituents of cellular structures and energy metabolism but also serve as key determinants of leaf elasticity, toughness, oiliness, and filling capacity. Elevated lipid content promotes denser tissue architecture and enhanced cell wall flexibility, reducing fragmentation during curing and aging, thereby maintaining superior appearance quality and processability (Li et al., 2026). During combustion, lipids undergo oxidation, pyrolysis, and rearrangement, yielding volatile compounds such as aldehydes, ketones, esters, and short-chain fatty acids. These pyrolysis products shape the aromatic profile of the smoke and interact synergistically with carbohydrates and nitrogenous compounds via Maillard reactions, thereby modulating flavor complexity and sensory perception (Peng et al., 2025). Moderate lipid accumulation favors a sensory profile characterized by a full-bodied aroma, delicacy, mildness, and a clean aftertaste. In contrast, insufficient lipid levels result in a thin aroma and harsh taste, whereas excessive accumulation may induce off-flavors or irritation (Xu et al., 2025).

#### 3.4.5 Volcano plot of differential metabolites

Comparative metabolomic analysis between high- and low-oil tobacco leaves revealed marked metabolic disparities (**Figure 9**). A total of 50 differentially accumulated metabolites (DAMs) were identified, comprising 23 up-regulated and 27 down-regulated metabolites in high-oil leaves. High-oil leaves generally exhibited enhanced glycolysis and lipid biosynthesis. Key intermediates, specifically glucose 1-phosphate and the signature lipid constituent oleic acid, were significantly enriched, supplying sufficient energy and precursors for oil accumulation. Concurrently, levels of characteristic secondary metabolites, such as limonin, increased in high-oil leaves, whereas melatonin, baicalein, and specific phenylpropanoid derivatives were notably down-regulated. This pattern reflects a distinct metabolic trade-off between secondary defense metabolism and lipid biosynthesis. In summary, the concurrent upregulation of oleic acid closely aligns with the relative oiliness index phenotype. Thus, oleic acid represents a potential biomarker for the discrimination and screening of high-oil tobacco, facilitating the elucidation of the molecular mechanisms governing oil formation (Annevelink et al., 2023; Yan et al., 2023).

#### 3.4.6 Box plot analysis of differential metabolites

**Figure 10:**
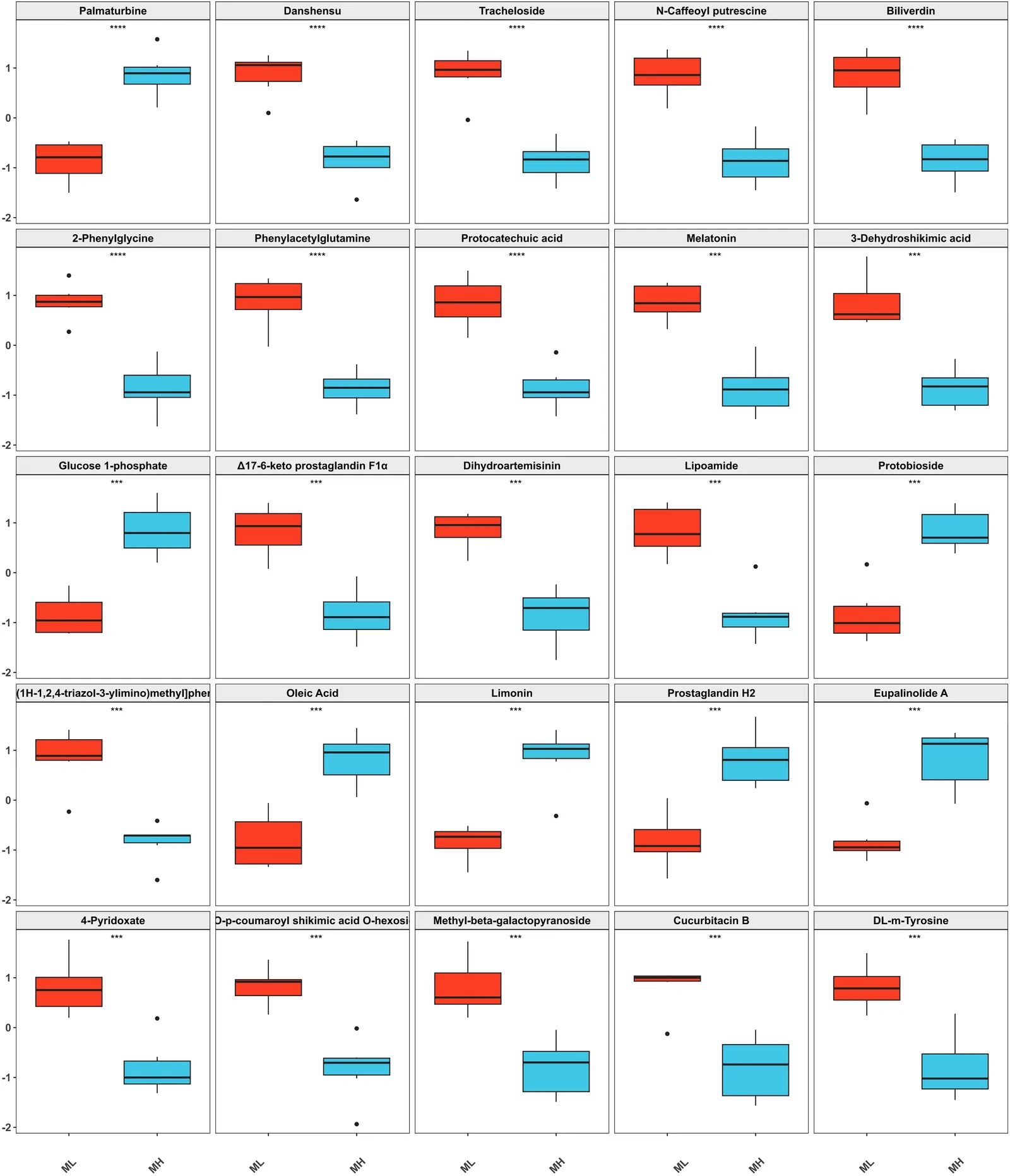
Box plot analysis of differential metabolites. MH: High-oil fresh tobacco leaves; ML: Low-oil fresh tobacco leaves. Data are presented as the mean ± standard deviation; ***P < 0.001; ***P < 0.001, compared with the control group.

To visualize intergroup metabolic variations, box plots were constructed for the top 25 representative differential metabolites identified via univariate analysis (**Figure 10**). The metabolic profiles of high-oil (MH) and low-oil (ML) fresh tobacco leaves exhibited significant divergence. In the MH group, the relative abundances of lipid metabolites defining the high-oil phenotype and sugar metabolic intermediates (e.g., glucose 1-phosphate) were markedly elevated. Conversely, the ML group displayed higher levels of phenolic acids, amino acid derivatives, and specific alkaloids. The pronounced accumulation of glucose 1-phosphate in MH leaves indicates active photosynthetic assimilation and an abundant supply of energy and carbon skeletons, thereby underpinning the high-oil phenotype. Furthermore, oleic acid exhibited substantial enrichment. As a pivotal unsaturated fatty acid, oleic acid not only maintains membrane fluidity but also acts as a key precursor for aroma generation during post-curing ripening. This metabolic signature aligns with the superior traits of high-oil tobacco, specifically accounting for its elevated relative oiliness index and vigorous glandular trichome secretion. Although HPLC quantification revealed significant differences in the levels of cembratriendiol, methyl nonanoate, and methyl palmitate between the high-oil and low-oil groups, these compounds were not annotated as differential metabolites in untargeted LC-MS/MS profiling. This discrepancy can be primarily attributed to inherent technical biases between analytical platforms, which precluded the effective detection of these specific metabolites under the conditions employed for non-targeted LC-MS/MS.

#### 3.4.7 Enrichment analysis of differential metabolites

**Figure 11:**
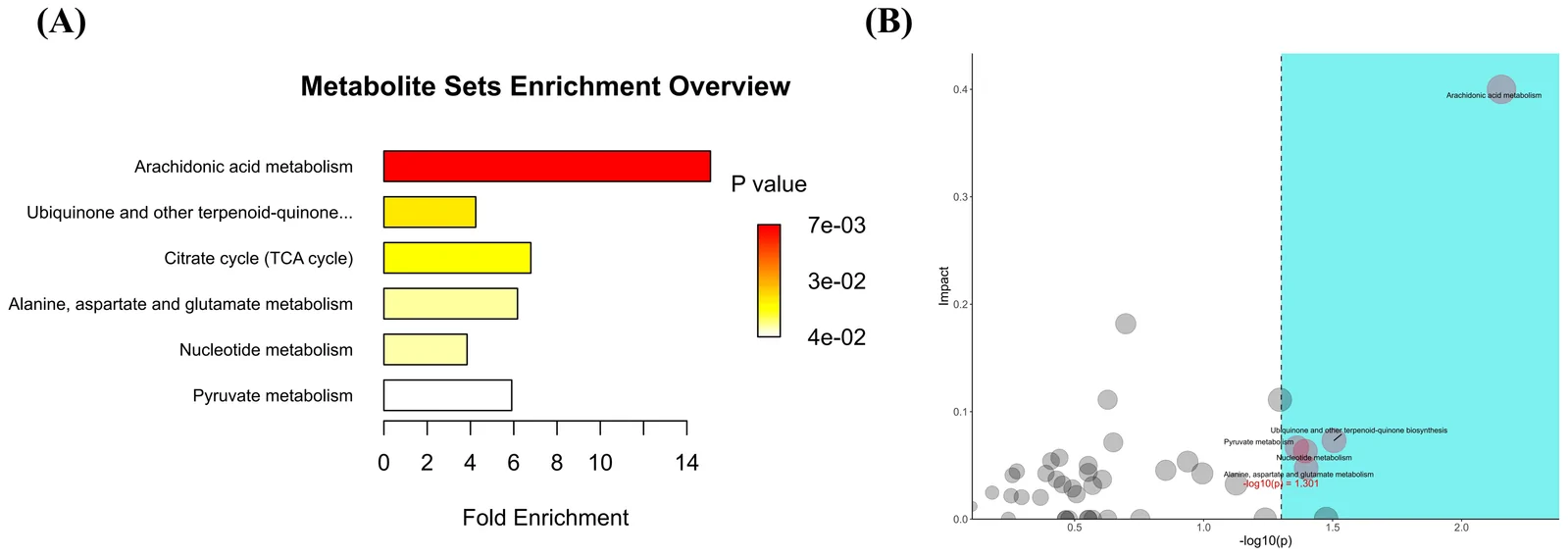
Enrichment **(A)** and topological **(B)** analyses of differential metabolites.

To elucidate metabolic disparities between high- and low-oil tobacco leaves, a metabolite set enrichment analysis (MSEA) was performed on the untargeted metabolomic data. As shown in **Figure 11**, differential metabolites were significantly enriched in multiple key primary and secondary metabolic pathways. Notably, arachidonic acid metabolism exhibited the highest enrichment score. Furthermore, significant enrichment was observed in central carbon and amino acid metabolism, including the tricarboxylic acid (TCA) cycle, alanine, aspartate and glutamate metabolism, and pyruvate metabolism. This suggests that high-oil leaves exhibit elevated metabolic flux and a distinct carbon–nitrogen allocation strategy. In summary, the development of high-oil tobacco is closely associated with metabolic processes that supply lipid precursors.

This study compared high- and low-oil-cake cultivation regimes and demonstrated the pronounced effects of oil-cake-based organic fertilizers on the cytological traits and metabolomic profiles of tobacco leaves. Nevertheless, certain limitations in the experimental design warrant attention. Specifically, the high-oil treatment differed from the low-oil treatment not only in oil-cake application rate but also in total nitrogen input (6.0 *vs*. 6.5 kg per thousand plants). Therefore, the observed differences in cellular architecture and metabolite accumulation should be interpreted as the integrated outcome of distinct cultivation practices rather than attributed exclusively to oil content per se. Future research should adopt controlled fertilization trials that maintain consistent nitrogen levels while modulating only the proportion of oil-cake amendments. Such an approach will help elucidate the specific regulatory mechanisms of lipid components or oil-cake-derived secondary metabolites during tobacco growth and development.

## 4 Conclusions

Microscopic and ultrastructural analyses of fresh Yunyan 87 tobacco leaves revealed a significantly higher density of glandular trichomes in high-oil leaves compared to low-oil counterparts. Specifically, trichomes on high-oil leaves were predominantly short-stalked, exhibiting markedly swollen heads and abundant secretions, coupled with relatively sparse cuticular ornamentation. Furthermore, high-oil leaves contained substantially more starch grains and osmiophilic granules, thereby promoting the formation and accumulation of aroma compounds. Additionally, cembratrienediol, a key trichome secretion, was significantly elevated in high-oil leaves. Similarly, lipid-related volatiles, such as methyl nonanoate and methyl palmitate, were markedly increased. Metabolomic profiling further confirmed the significant enrichment of differential metabolites in the arachidonic acid metabolism pathway. The concurrent upregulation of the characteristic lipid component, oleic acid, closely aligned with the oil content phenotype, supporting its potential as a biomarker for identifying high-oil tobacco. This study provides a theoretical basis for the targeted breeding and agronomic management of high-oil tobacco cultivars.

## Data Availability

The original contributions presented in the study are included in the article/**Supplementary Material**. Further inquiries can be directed to the corresponding author.
