# Supplementary material for: Cytological basis and key metabolite profiling of fresh tobacco leaves with different oiliness indices in Yunyan 87
Source: Front Plant Sci. 2026 Sep 4;17:1911537. doi: 10.3389/fpls.2026.1911537 (PMC13585907; doi:10.3389/fpls.2026.1911537)
Supplement: Supplementary file 1 [file DataSheet1.pdf]

Supplementary Material

1.1 Supplementary Figures

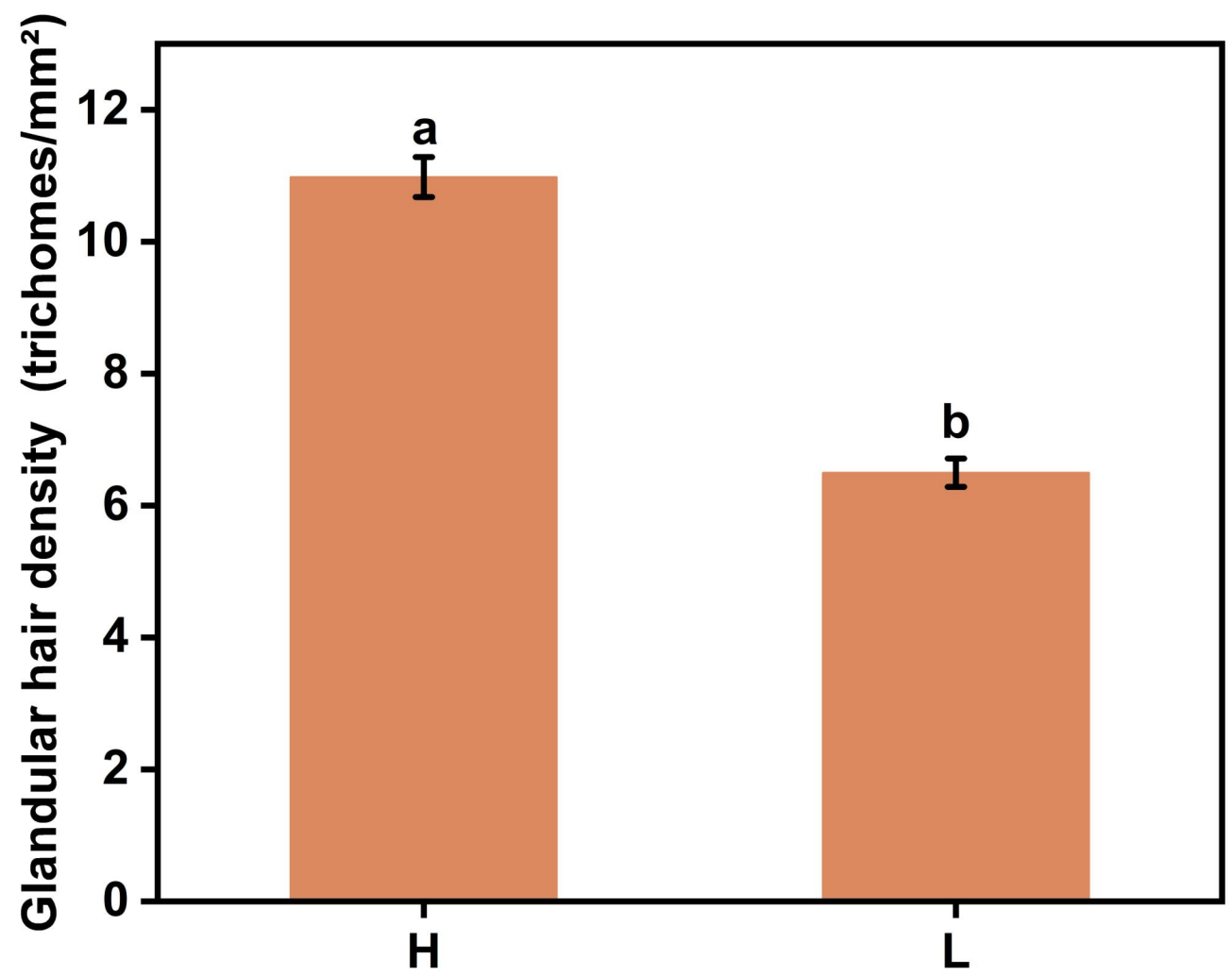

Supplementary Figure 1. Glandular trichome density in tobacco leaves with different oil contents. H, High-oil fresh leaves; L, Low-oil fresh leaves. Mean values followed by different lowercase letters are significantly different at $P < 0.05$ based on one-way ANOVA with *Duncan*'s multiple range test.

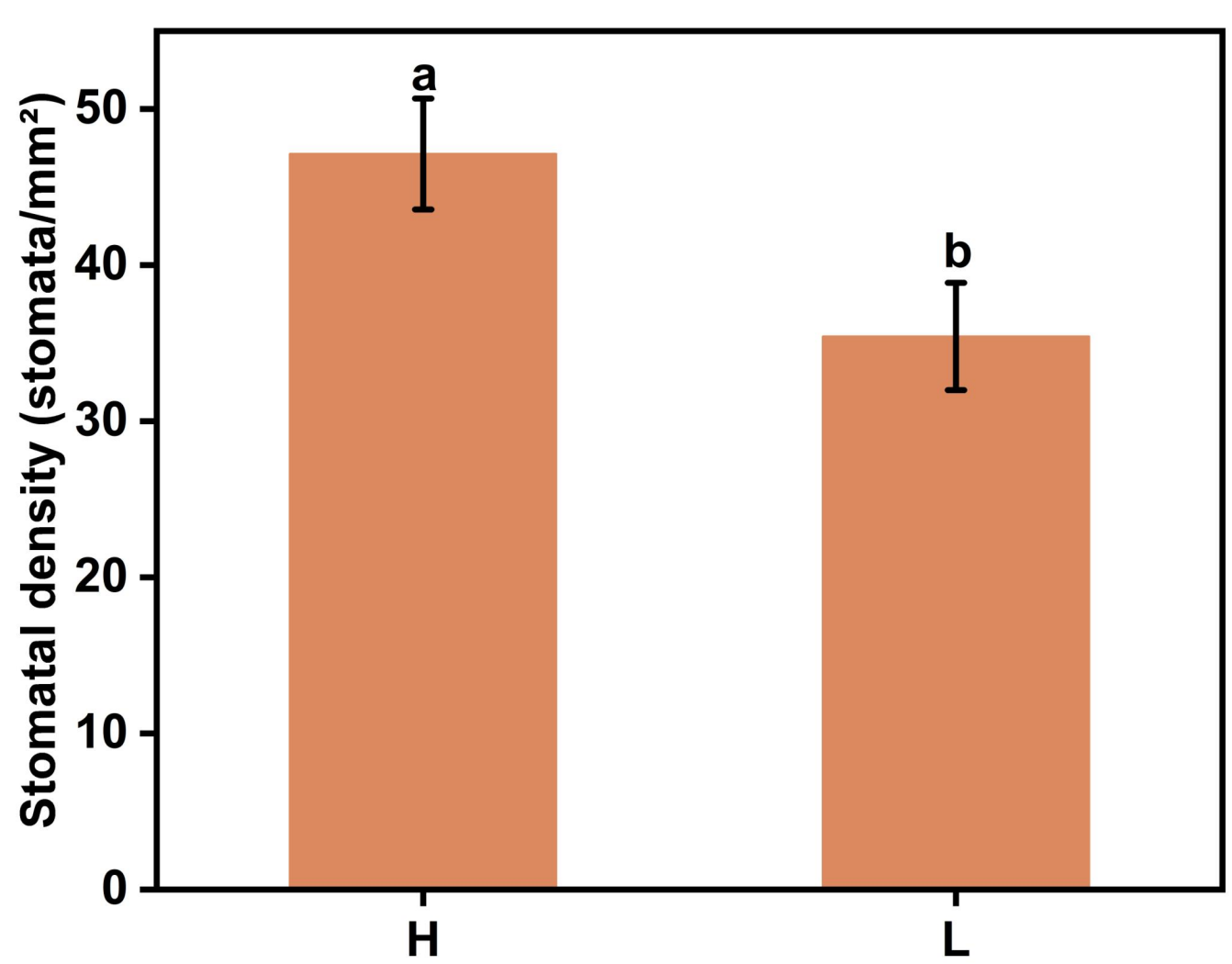

Supplementary Figure 2. Stomatal density in tobacco leaves with different oil contents. H, High-oil fresh leaves; L, Low-oil fresh leaves. Mean values followed by different lowercase letters are significantly different at $P < 0.05$ based on one-way ANOVA with *Duncan*'s multiple range test.

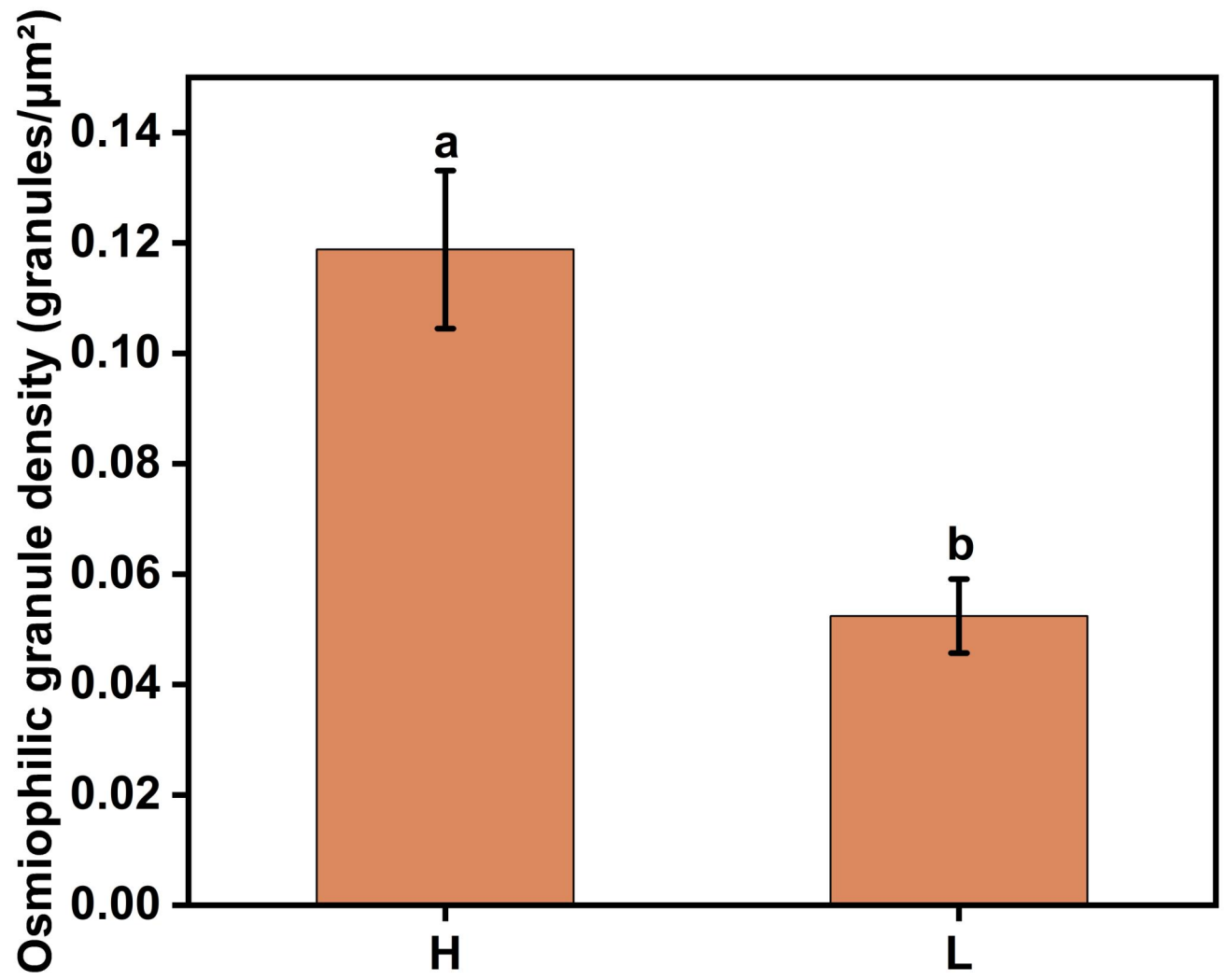

Supplementary Figure 3. Osmiophilic particle density in tobacco leaves with different oil contents. H, High-oil fresh leaves; L, Low-oil fresh leaves. Mean values followed by different lowercase letters are significantly different at $P < 0.05$ based on one-way ANOVA with *Duncan*'s multiple range test.

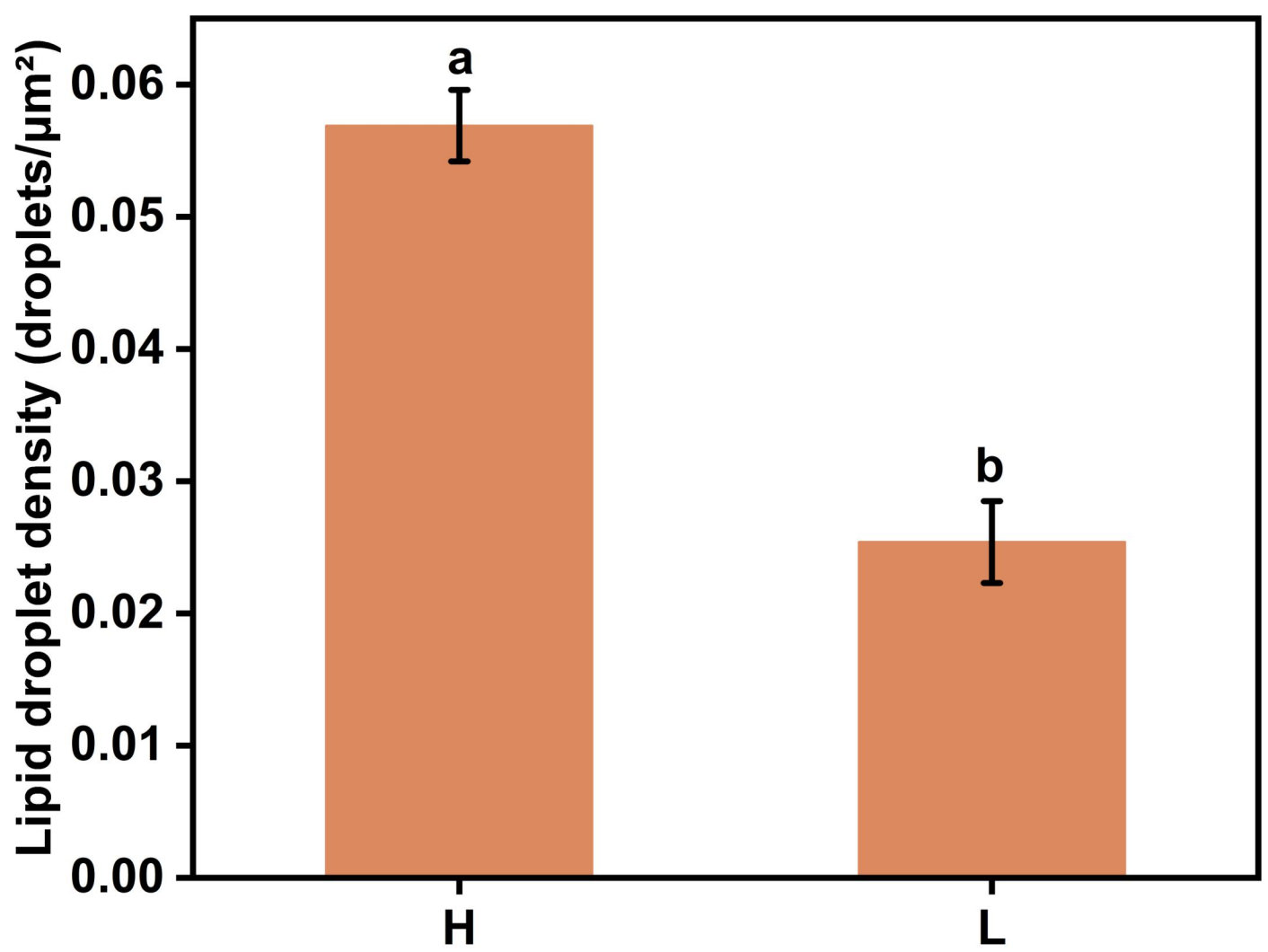

Supplementary Figure 4. Lipid droplet density in tobacco leaves with different oil contents. H, High-oil fresh leaves; L, Low-oil fresh leaves. Mean values followed by different lowercase letters are significantly different at $P < 0.05$ based on one-way ANOVA with *Duncan*'s multiple range test.

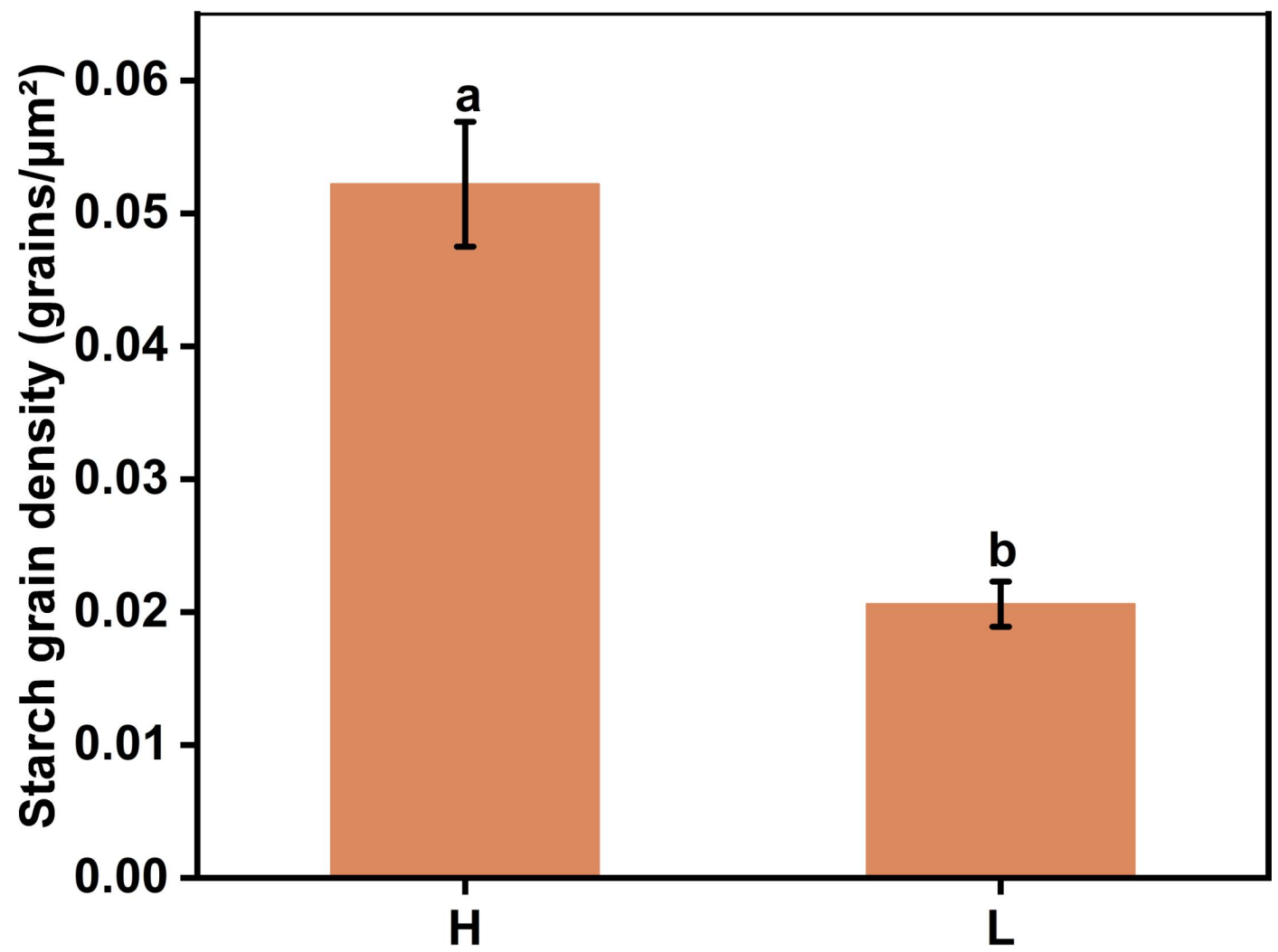

Supplementary Figure 5. Starch grain density in tobacco leaves with different oil contents. H, High-oil fresh leaves; L, Low-oil fresh leaves. Mean values followed by different lowercase letters are significantly different at $P < 0.05$ based on one-way ANOVA with *Duncan*'s multiple range test.
