# Supplementary material for: Cytological basis and key metabolite profiling of fresh tobacco leaves with different oiliness indices in Yunyan 87
Source: Front Plant Sci. 2026 Sep 4;17:1911537. doi: 10.3389/fpls.2026.1911537 (PMC13585907; doi:10.3389/fpls.2026.1911537)
Supplement: Supplementary file 2 [file Table1.docx]

| Supplementary Table 1. Precision and repeatability verification of relative oiliness indices detection pen in field in-situ measurement | | | | | | | | | | | | | |
| --- | --- | --- | --- | --- | --- | --- | --- | --- | --- | --- | --- | --- | --- |
| Leaf No. | Reading 1 | Reading 2 | Reading 3 | Reading 4 | Reading 5 | Reading 6 | Reading 7 | Reading 8 | Reading 9 | Reading 10 | Mean | SD | RSD(%) |
| H-1 | 52.3 | 53.4 | 52.8 | 52.6 | 53.4 | 52.4 | 53.2 | 52.1 | 52.3 | 52.9 | 52.74 | 0.48 | 0.9 |
| H-2 | 51.6 | 52.2 | 51.4 | 52.2 | 51.7 | 52.4 | 51.3 | 52.5 | 51.7 | 52.1 | 51.91 | 0.42 | 0.81 |
| H-3 | 53.2 | 52.9 | 53.3 | 52.6 | 54.2 | 52.8 | 53.3 | 52.1 | 53.9 | 52.3 | 53.06 | 0.66 | 1.25 |
| H-4 | 53.2 | 53.2 | 54.4 | 53.1 | 52.9 | 53.2 | 52.5 | 53.3 | 53.2 | 52.9 | 53.19 | 0.49 | 0.91 |
| H-5 | 51.8 | 52.3 | 51.6 | 52.3 | 53.2 | 51.4 | 52.3 | 51.4 | 52.2 | 52.7 | 52.12 | 0.58 | 1.11 |
| H-6 | 52.6 | 53.2 | 52.3 | 53.7 | 52.1 | 53.2 | 52.8 | 53.3 | 52.4 | 53.9 | 52.95 | 0.61 | 1.14 |
| M-1 | 50.3 | 49.8 | 50.6 | 49.7 | 50.4 | 51.5 | 50.8 | 49.8 | 50.5 | 49.6 | 50.30 | 0.59 | 1.18 |
| M-2 | 49.4 | 50.6 | 49.7 | 50.4 | 49.8 | 50.6 | 49.3 | 50.2 | 49.4 | 50.2 | 49.96 | 0.50 | 1.01 |
| M-3 | 50.4 | 50.8 | 51.2 | 50.6 | 50.7 | 50.4 | 51.4 | 50.8 | 51.4 | 50.5 | 50.82 | 0.38 | 0.75 |
| M-4 | 50.7 | 49.2 | 50.3 | 50.2 | 50.4 | 51.2 | 50.8 | 49.9 | 50.2 | 50.4 | 50.33 | 0.54 | 1.07 |
| M-5 | 49.5 | 50.6 | 49.1 | 49.8 | 50.5 | 49.8 | 50.2 | 49.4 | 50.6 | 49.1 | 49.86 | 0.59 | 1.18 |
| M-6 | 50.3 | 50.6 | 49.7 | 50.2 | 50.9 | 49.7 | 50.6 | 50.7 | 50.6 | 50.4 | 50.37 | 0.41 | 0.81 |
| S-1 | 47.1 | 46.6 | 47.4 | 47.7 | 46.3 | 47.8 | 46.5 | 47.3 | 47.1 | 46.4 | 47.02 | 0.54 | 1.16 |
| S-2 | 48.4 | 47.3 | 48.4 | 47.9 | 47.3 | 48.1 | 47.6 | 48.5 | 47.4 | 47.1 | 47.80 | 0.53 | 1.10 |
| S-3 | 47.7 | 47.4 | 48.3 | 47.6 | 46.1 | 47.3 | 47.5 | 47.6 | 46.9 | 47.3 | 47.37 | 0.57 | 1.21 |
| S-4 | 46.4 | 47.6 | 46.3 | 46.2 | 47.7 | 46.7 | 47.4 | 46.1 | 46.5 | 46.3 | 46.72 | 0.61 | 1.31 |
| S-5 | 46.8 | 47.5 | 46.2 | 47.1 | 46.6 | 47.3 | 46.4 | 47.2 | 46.9 | 47.0 | 46.90 | 0.41 | 0.87 |
| S-6 | 48.4 | 47.7 | 48.4 | 48.6 | 47.4 | 48.3 | 47.5 | 48.2 | 47.8 | 48.0 | 48.03 | 0.41 | 0.86 |
| L-1 | 43.4 | 43.8 | 44.3 | 43.5 | 44.7 | 43.1 | 44.3 | 43.9 | 44.5 | 43.6 | 43.91 | 0.52 | 1.19 |
| L-2 | 44.3 | 43.8 | 44.5 | 44.1 | 43.8 | 44.5 | 43.3 | 44.9 | 44.6 | 44.1 | 44.19 | 0.47 | 1.06 |
| L-3 | 43.1 | 43.6 | 42.8 | 43.3 | 43.9 | 43.6 | 43.4 | 42.6 | 43.4 | 43.6 | 43.33 | 0.39 | 0.92 |
| L-4 | 44.3 | 44.6 | 43.4 | 44.3 | 43.8 | 44.4 | 43.9 | 44.3 | 44.5 | 43.7 | 44.12 | 0.39 | 0.89 |
| L-5 | 42.7 | 43.4 | 42.7 | 43.1 | 42.8 | 43.8 | 43.0 | 42.6 | 43.7 | 42.8 | 43.06 | 0.43 | 1.00 |
| L-6 | 44.4 | 44.6 | 43.5 | 44.5 | 44.6 | 43.2 | 44.5 | 44.4 | 43.8 | 44.3 | 44.18 | 0.49 | 1.13 |

Note: "High oil (H)", "Medium oil (M)", "Slight oil (S)", and "Low oil (L)"
